# Choose Your Maternal DNA Wisely: Intrinsic Exercise Capacity and Mitochondrial Genome Influence Vascular Function in Rats

**DOI:** 10.1093/function/zqaa039

**Published:** 2020-12-28

**Authors:** Austin T Robinson

**Affiliations:** Neurovascular Physiology Laboratory, School of Kinesiology, Auburn University, Auburn, AL 36849, USA

## A Perspective on “Intrinsic exercise capacity and mitochondrial DNA lead to opposing vascular-associated risks”

“Choose your parents wisely” is a popular adage coined by the British Philosopher Bertrand Russell, and often used when discussing issues such as socioeconomic position and genetic contributions to disease risk. When it comes to vascular function, a predictor of cardiovascular disease risk, the recent study in *Function* by Roy et al.^1^ extends the adage to “choose our maternal mitochondrial DNA wisely.” The study characterized vascular function in rats with a low capacity for running (LCR) and high capacity for running (HCR). Aerobic exercise is a positive health behavior for the prevention and treatment of cardiovascular and metabolic disease states. Aerobic exercise training generally leads to improved cardiorespiratory fitness (CRF),^2^ but genetic predispositions for intrinsic capacity and trainability lead to substantial variability.^3^ Importantly, CRF is a predictor of cardiovascular and all-cause mortality.^4^ Moreover, resistance artery dysfunction precedes end-organ damage from hypertension and cardiovascular disease.^5^ Thus, the authors sought to determine whether CRF influences resistance artery structure and function, cardiac function, perivascular adipose tissue (PVAT), and bioenergetic profiling in vascular cells. Moreover, the researchers sought to determine whether the inherited mitochondrial genome associated with intrinsic exercise capacity also independently influences vascular physiology. 

The investigators studied rats artificially selected (within-family) for intrinsic aerobic endurance running capacity to generate LCR and HCR male rats.^3^ As previously described,^6^ the authors also generated conplastic strains, whereby LCR male rodents were bred with mitochondrial DNA (mtDNA) of female HCR rodents (LCR-mt^HCR^) and vice versa (HCR-mt^LCR^). Specifically, HCR female offspring were backcrossed with male LCR (or the reciprocal) via inbreeding, and this backcross procedure was repeated over several generations to generate the LCR-mt^HCR^ and HCR-mt^LCR^. The investigators performed echocardiography to assess cardiac function and left ventricular mass, wire, and pressure myography to assess arterial vasodilatory function (with and without PVAT) and mechanics, macroscopic tissue imaging of PVAT, and bioenergetic assays with vascular smooth muscle cells (VSMCs).

**Figure 1.**
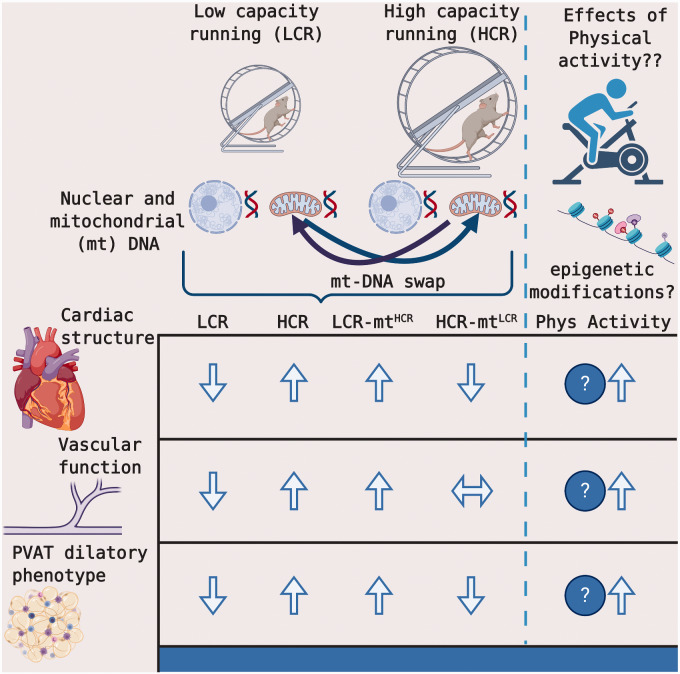
A Summary of the Key Findings from Roy et al. and an Interesting Future Direction. Male rats were artificially selected for LCR and HCR. Conplastic strains were developed to transpose the mitochondrial (mt) DNA of HCR rats onto LCR rats (LCR-mt^HCR^) and vice versa (HCR-mt^LCR^). The LCR mt DNA was associated with reduced left ventricular mass, small artery vascular dysfunction, and provasoconstrictive PVAT phenotype, whereas HCR mt DNA was associated with greater left ventricular mass, preserved small artery vascular function, and a provasodilatory perivascular PVAT phenotype. An interesting future direction will be to determine whether regular physical activity (eg, voluntary or forced running) can prevent or attenuate many of the negative vascular consequences of being born with the LCR phenotype, similar to the LCR-mt^HCR^ rats. Figure created with Biorender.com.

Compared to HCR, LCR rats had higher body mass, epididymal fat mass, and blood pressure. Regarding cardiac measures, HCR rats presented higher left ventricular mass than LCR rats (see Figure 1). Compared to LCR, HCR rats exhibited lower relative ventricular wall thickness and fractional area change, a surrogate of systolic function, but no differences were observed for multiple measures of cardiac output, velocity of circumferential fiber shortening, or myocardial contractility. Interestingly, mitochondrial swap increased left ventricular mass in LCR (ie, ↑ in LCR-mt^HCR^) but decreased left ventricular mass and several indices of cardiac performance in HCR-mt^LCR^ relative to HCR.

The key findings were that compared to HCR, LCR rats presented with lower endothelium-dependent (acetylcholine) and endothelium-independent (sodium nitroprusside) vasodilation in mesenteric resistance arteries. Mitochondrial swap rescued endothelium-dependent and endothelium-independent vasodilation in LCR (↑ in LCR-mt^HCR^), however, it did not reduce vascular function in HCR-mt^LCR^. Using mesenteric arterioles and plotting internal lumen and external diameters with intraluminal pressure curves, the authors revealed that the arterioles from LCR rats exhibited hypotrophic remodeling (ie, smaller arterioles with smaller vascular wall thickness). Moreover, mitochondrial swap did not have an effect on either the LCR or HCR phenotype.

Additional findings included that, on aggregate, the mesenteric PVAT of HCR rats exhibited a ridged, bulbous appearance, but bulbous fat was not present in LCR rats, nor the mitochondrial swapped rats. Importantly, PVAT modulates vascular contractility and arterial pressure due to paracrine activity influencing inflammation and the redox environment of adjacent vessels.^7^ Interestingly, when the authors performed “sandwich bioassays” with strips of isolated PVAT around the resistance arteries, LCR arteries sandwiched with HCR PVAT presented significantly improved endothelium-dependent vasodilation. Conversely, HCR arteries sandwiched with LCR PVAT presented with reduced vasodilation. Lastly, no differences existed in multiple measures of mitochondrial respiration in VSMCs between LCR and HCR rodents, apart from LCR VSCM exhibiting increased nonmitochondrial respiration. However, it was unclear whether the difference in nonmitochondrial respiration was consequential regarding reactive oxygen species (ROS) production or nitric oxide (NO) bioavailability.

The authors concluded that cardiac structure and vasodilator experiments suggest a physiologically relevant interplay between the nuclear genome and the maternally inherited mitochondrial genome. Specifically, high intrinsic exercise capacity is a significant factor for greater vascular function. The conclusion was supported by the findings that LCR-mt^HCR^ recused vascular function, and PVAT from HCR rats also improved endothelium-dependent vasodilation in LCR rats. It is plausible that intrinsic CRF would influence both adipose tissue phenotype and the vasculature, which play an integral role in substrate availability and delivery, as key factors in exercise capacity.

This eloquent study paves the way for future investigations to elucidate several remaining important questions, such as (1) to what extent intrinsic exercise capacity and mitochondrial DNA influence vascular function in female and older rodents, (2) if the reduced endothelium-dependent vasodilation in LCR rats is mediated by reduced NO synthesis and/or bioavailability or a combination of NO and/or other vasodilators (eg, endothelium-dependent hyperpolarizing factor(s) or prostacyclin), (3) the role of mitochondrial ROS and other vascular sources of ROS (eg, NADPH oxidase and xanthine oxidase) in contributing to the vascular phenotypes associated with HCR and LCR, and (4) determining whether exercise, similar to mitochondrial swapped conplastic strains, can also rescue endothelial dysfunction in LRC rodents (Figure 1).

At the population level, we cannot do much to improve an individual’s intrinsic CRF. Indeed, the HERITAGE family study established that intrinsic capacity and trainability are genetically heritable in humans.^8^ However, recent studies emphasize the role of varying combinations of exercise modalities, intensities, and frequencies, and environmental factors, (eg, sleep, diet, and social support) to promote exercise adaptation and the subsequent health benefits.^2^^,^^9^ Thus, from a public health standpoint, we should implement evidence-based strategies to increase physical activity in activities of daily living and remove barriers to exercise.^10^ Nonetheless, from a physiology standpoint, this study provides valuable novel information pertaining to how intrinsic CRF protects against cardiovascular disease, specifically by mediating resistance artery function, in part via perivascular adipose and the surrounding paracrine milieu. Moreover, the protective effect of the mt^HCR^ on LCR rodents demonstrates that mitochondrial DNA is an essential oddment to the protective phenotype conferred by high CRF. These findings will hopefully pave the way for exciting future research to determine mitochondrial-based targets to help prevent and treat cardiovascular disease.
